# Aptamer Functionalized Upconversion Nanotheranostic Agent With Nuclear Targeting as the Highly Localized Drug-Delivery System of Doxorubicin

**DOI:** 10.3389/fbioe.2021.639487

**Published:** 2021-02-22

**Authors:** Xinyue Song, Tao Yan, Feng Tian, Fengyan Li, Linlin Ren, Qiong Li, Shusheng Zhang

**Affiliations:** ^1^Shandong Provincial Key Laboratory of Detection Technology for Tumor Markers, College of Chemistry and Chemical Engineering, Linyi University, Shandong, China; ^2^Materials Science and Engineering, Mobile Postdoctoral Center, Qingdao University, Shandong, China

**Keywords:** aptamers, doxorubicin, drug delivery, nuclear targeting, upconversion nanotheranostic agent

## Abstract

As a widely used anticancer drug, doxorubicin (DOX) could induce cell death mainly *via* interfering with DNA activity; thus, DOX could perform therapeutic effects mainly in the cell nucleus. However, most of the reported drug delivery systems lacked the well localization in the nucleus and released DOX molecules into the cytoplasm. Due to formidable barriers formed in the nuclear envelope, only around 1% of DOX could reach the nucleus and keep active. Therefore, DOX molecules were inevitably overloaded to achieve the desired therapeutic efficacy, which would induce serious side effects. Herein, we developed a highly localized drug nanocarrier for *in situ* release of DOX molecules to their action site where they could directly interfere with the DNA activity. In this work, we used cationic polymer-modified upconversion nanoparticles (UCNPs) as the luminescence core and gene carrier, while aptamers served as the DNA nanotrain to load DOX. Finally, the prepared nanotheranostic agent displayed good targetability, high cell apoptosis ratio (93.04%) with quite lower concentration than the LC50 of DOX, and obvious inhibition on tumor growth.

## Introduction

Nowadays, cancer has been a major human health problem although great progress was made in its diagnosis and treatment (Skrott et al., 2017; Wu et al., 2019). Among the conventional therapeutic strategies, chemotherapy remains as one of the most commonly used techniques to treat cancers in the clinic (Langley and Rothwell, 2013; Matera et al., 2018). As a widely used anticancer drug, doxorubicin (DOX) could fight against various cancers. However, its short half-life, obvious drug resistance, and serious adverse effects have been main bottlenecks for its clinical applications (Carvalho et al., 2009). Recently, smart drug delivery systems have been developed as promising strategies to selectively deliver chemotherapeutic drugs to tumor cells and achieve targeted cancer therapy. In these nanocarriers, DOX molecules were usually released due to the trigger of the tumor microenvironment including acidity, hypoxia, reductive potential, and overexpressed enzymes or external assisted stimuli such as light, ultrasound, magnetic field, and temperature (Abouelmagd et al., 2014). DOX could fight against various cancers *via* multiple interaction mechanisms including intercalating into DNA, covalently binding to proteins involved in DNA replication and transcription, or inhibiting topoisomerase II (Wagstaff and Jans, 2009). Thus, the cell nucleus is the main reaction place where DOX molecules would elicit their pharmacological effects. So the drug delivery system with effective nuclear targeting was expected to show enhanced therapeutic efficacy (Pan et al., 2012). However, most of the reported drug delivery systems have been designed to distribute in the cytoplasm. The released DOX molecules would diffuse into the nucleus only *via* the passive diffusion (Sui et al., 2011; Dai et al., 2016). Usually, the nuclear envelope formed a formidable barrier and only let around 1% of macromolecules or drug molecules pass through. In addition, inhibited by various biobarriers, free DOX molecules were quite difficult to keep active after arriving at the nucleus (Kim et al., 2010). Therefore, DOX molecules were inevitably overloaded to achieve the desired therapeutic efficacy, which would induce serious side effects. Thus, it is urgent to develop novel drug delivery systems for the direct delivery of DOX molecules to their action sites.

In addition, the use of single drug molecules often fails to achieve complete cancer remission. Many studies represent that combination chemotherapy which co-administered two drug molecules leads to significantly greater activity than single chemotherapy (Lee et al., 2010; Shanmugam et al., 2014). Inspired by these studies, we tried to explore a novel combination therapy by using drug molecules and DNA strands. AS1411 is a selected aptamer showing specific binding to nucleolin, a protein overexpressed in the nucleus and cellular membrane of cancer cells (Teng et al., 2007; Xiao et al., 2012). Thus, the nucleolin/AS1411-mediated process is an effective strategy for targeting proliferating cancer cells and achieving the nucleus-localized drug delivery (Rosental et al., 2011). As a prominent cancer-associated protein, proliferating cell nuclear antigen (PCNA) is highly expressed in the nucleus of rapidly proliferating cancer cells and coordinates the essential cellular functions for cell growth, death, or maintenance since its trimmers could form a molecular sliding clamp around the DNA double helix to involve in the DNA replication and repair (Nagel et al., 2016). Therefore, it is proposed that PCNA could act as a potential non-oncogenic target for anticancer therapy (Stoimenov and Helleday, 2009). Strategies which prevent PCNA from binding with the PCNA-interacting proteins would be developed as effective anticancer therapies (Stoimenov and Helleday, 2009; Wang, 2014; Kowalska et al., 2017). In 2017, professor Wojciech Strzalka screened out an anti-PCNA aptamer using the systematic evolution of ligands by the exponential enrichment (SELEX) method and proved its potential role in interfering with PCNA bioactivities. The obtained anti-PCNA aptamer showed selective binding with PCNA, and the formed anti-PCNA aptamer/PCNA/DNA pol complex would effectively block the replication of the DNA template. Meanwhile, it could also inhibit the activity of human DNA polymerase δ and ε at nM concentrations (Zhang et al., 2014). Since DNA strands with sequential G–C base pairs would provide loading sites for DOX molecules *via* intercalating into the major or minor groove of the double-helix structure (Zhou et al., 2012), thus the anti-PCNA aptamer could be used to develop effective and feasible drug delivery systems for the *in situ* release of DOX molecules to their exact action sites, which have not been investigated before.

**SCHEME 1:**
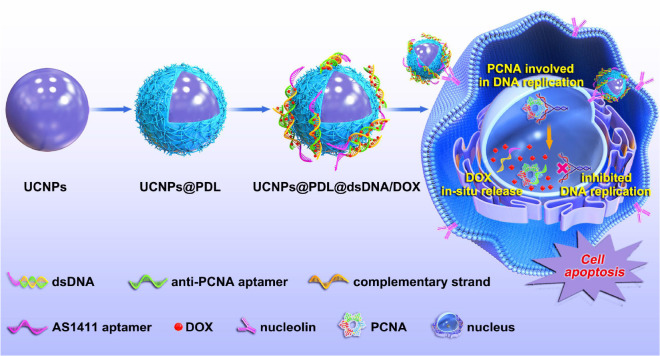
Procedure of the designed UCNPs@PDL@dsDNA/DOX nanotheranostic agent as the highly localized drug-delivery system.

Upconversion nanoparticles (UCNPs) are special fluorescent materials which could convert continuous near infrared (NIR) excitation waves to ultraviolet-visible or NIR emissions based on the two-photon or multiphoton mechanism (Liu et al., 2013). Prior to conventional fluorescent dyes, UCNPs exhibit characteristic luminescent properties including tunable emission bands, large anti-Stokes shift, long lifetime, good photostability, low toxicity, no photobleaching, and no blinking (Haase and Schafer, 2011). Furthermore, NIR as the excitation light of UCNPs is coincident with the biological transparency window; thus, greater tissue penetration depth and reduced background autofluorescence would be obtained (Dong et al., 2017). Therefore, UCNPs have shown outstanding merits in *in vitro* fluorescence detection (Tian et al., 2015), multimodal imaging in small animals, and drug carriers (Vasiliu et al., 2017). In this work, we used UCNPs as the luminescence core and then coated them with a layer of cationic polymer, poly-D-lysine (PDL), as a gene carrier which has been frequently used due to its low gene transfection and the easy binding with nucleic acids (Song et al., 2017). Anti-PCNA aptamer and AS1411 aptamer were used as the DNA nanotrain to load DOX molecules. As expected, the obtained UCNPs@PDL nanoprobe would carry DNA nanotrains to selectively target cancer cells and then locate in the nucleus where the anti-PCNA aptamer would specially bind with PCNA to induce the *in situ* release of DOX molecules. Thus, the released free DOX and inhibited PCNA would directly interfere with DNA replication and then cooperatively induce cell apoptosis (Scheme 1). As shown in the cell and animal experiment, the developed drug delivery system would induce around 93% of cell apoptosis with only 0.45 μg/mL of DOX molecules without obvious drug resistance and further greatly inhibit the tumor growth with negligible systematic toxicity to normal tissue. Therefore, the constructed UCNPs@PDL@dsDNA/DOX nanotheranostic agent could fully perform the synergistic effect of anti-PCNA aptamer and DOX molecules and achieve the targeted location therapy to obtain satisfactory therapeutic efficacy.

## Experimental Section

### Preparation of the UCNPs

UCNPs were prepared based on a layer-by-layer seed-mediated shell growth strategy (Li et al., 2015a; Song et al., 2018; Yue et al., 2018). Based on the previously reported methods (Chaires et al., 1990; Li et al., 2015a), Y(oleate)_3_ (0.075 mmol/L) and Ln(oleate)_3_ (0.05 mmol/L, Y:Yb:Tm:Ho = 77.8:20:0.2:2 in mol) were prepared in oleic acid (OA)/1-octadecene (ODE) mixing solution (v/v = 1:1). To prepare the NaYF_4_ core, 13.0 mL Y(oleate)_3_ solution and 0.84 g NaF were mixed in 7.0 mL OA/ODE mixing solvent (v/v = 1:1), reacted at 110°C for 1.0 h under the protection of argon (Ar), and then further reacted at 340°C for another 2.0 h. Then, 8.0 mL Ln(oleate)_3_ solution was injected into the above solution and reacted at 340°C for 20 min to grow the luminescent shell on the surface of the NaYF_4_ core. Finally, 8.0 mL Y(oleate)_3_ solution was followed, added, and reacted for another 20 min to prepare the outer shell NaYF_4_. The prepared UCNPs were precipitated in twofold volume of ethanol, centrifugally collected, and washed with hexane/ethanol (v/v = 1:6) for several times. The final oleic acid-protected UCNPs were dispersed in hexane and stored at −20°C for further use.

### Preparation of the UCNPs@PDL Nanoprobe

Firstly, the surface ligand of the prepared UCNPs was removed with the acid-treatment method (Chaires et al., 1990; Li et al., 2015b). Briefly, 20.0 mg of the protected UCNPs was dispersed in 30.0 mL acidic ethanol solution (pH = 1.0) and reacted under ultrasonification. After 1.0 h, the bared UCNPs could be collected by centrifugation, washed with ethanol for several times, and then stored in ultrapure water. To further coat the cationic polymer, bared UCNPs (5.0 mg) were mixed with PDL aqueous solution (0.6 mg/mL, 2.5 mL), ultrasonicated for 10 min, and then stirred for overnight. The obtained UCNPs@PDL was washed with ultrapure water and redispersed in ultrapure water for further use.

### Formation of DNA Nanotrain

In this work, the designed DNA nanotrain contained the anti-PCNA aptamer as single DNA 1 while the anti-PCNA aptamer complementary strand contained the AS1411 aptamer as single DNA 2 (Table S1). The formed duplex has 31 G–C base pairs suitable for DOX loading.

### Preparation of the UCNPs@PDL@dsDNA/DOX Nanotheranostic Agent

DNA-1 (10 μM) was mixed with an equal molar amount of DNA-2 in TE buffer (10 mM Tris–HCl, 1.0 mM EDTA, 12.5 mM MgCl_2_, pH-8.0). The hybridization was achieved on a thermal cycler with a designed program (heat at 95°C for 5 min, cool to 25°C at a rate of 0.1°C/s, and keep at 25°C for 1.5 h at least). Then, 200 μL of the hybridized DNA duplex (dsDNA) was mixed with DOX aqueous solution (20.0 μL, 0.36 mg/mL) for 6.0 h to load DOX molecules. Finally, the obtained UCNPs@PDL solution (1.0 mg/mL, 1.0 mL) was mixed with the above dsDNA-DOX and vibrated for overnight to obtain the UCNPs@PDL@dsDNA/DOX nanotheranostic agent.

### Cell Culture

A549 cancer cells and L132 embryonic lung cells were cultured in Dulbecco’s modified Eagle’s medium (DMEM) containing 10% heat-inactivated fetal bovine serum (FBS), penicillin (100 U/mL), and streptomycin (100 U/mL) at 37°C in humidified air containing 5% CO_2_.

### Cellular Uptake and Localization

A549 cancer cells and L132 embryonic lung cells were transferred into glass coverslips, respectively, and cultured for 12 h. Then, the prepared UCNPs@PDL@dsDNA/DOX nanoprobe (90 μg/mL) was added and cells were incubated for different times. After removing the excess nanoprobe, cells were stained with the commercial fluorescent dye, the Lyso-Tracker Red, guided by the manufacturer’s instructions. Finally, the two-photon laser confocal scanning microscope was used to observe and record the fluorescence spectra of the corresponding cells. The upconversion luminescence channel was collected at 620–680 nm under excitation by a 980-nm laser while lysosome information was collected at 580–600 nm under excitation at 543 nm.

For further study, the release mechanisms of DOX, Cy5-labeled DNA-1, and BHQ-3-labeled DNA-2 were used to prepare the corresponding fluorescence-labeled UCNPs@PDL@dsDNA/DOX nanoprobe (Supplementary Table S1). The above fluorescence-labeled UCNPs@PDL@dsDNA/DOX nanoprobe (90 μg/mL) was used to incubate cells on the glass coverslips for 24 h. After removing the excess nanoprobe, the cell nucleus was stained with the commercial Hoechst 33,342 fluorescent dye. Then, fluorescence information was recorded at different channels with the laser confocal scanning microscope. Briefly, fluorescence information of the nucleus was recorded at the blue channel (410–460 nm) under excitation at 405 nm. The fluorescence information of DOX molecules was recorded at the green channel (525–620 nm) under excitation at 488 nm. The fluorescence information of the Cy5-labeled anti-PCNA aptamer was recorded at red channel (640–720 nm) under excitation at 633 nm.

### Efficacy Assay in Living Cells

The efficacy assay of the prepared nanotheranostic agent for living cells was evaluated *via* CCK-8 assay and flow cytometry. Firstly, CCK-8 assay was used to evaluate the *in vitro* cytotoxicity of free DOX molecules and the prepared nanoprobes including UCNPs@PDL, UCNPs@PDL@dsDNA, and UCNPs@PDL@dsDNA/DOX. Briefly, A549 cancer cells and L132 embryonic lung cells were cultured in 96-well flat-bottom microtiter plates, respectively, at a density of 15,000 cells/well for 24 h. Afterward, different concentrations of the prepared nanoprobe (0–90 μg/mL) were added into the corresponding wells. After incubating the cells for a certain time, excess nanoprobe was washed away with PBS buffer. Then, 10 μL of CCK-8 agent was added into each well and the corresponding cells incubated for another 40 min. Finally, the microplate reader was used to record the absorbance of cells at 450 nm. The calculation of cell viability (%) was based on the below equation: Cell Viability(%) = (Mean Absorbance_treated wells_ – Mean Absorbance_blank wells_)/(Mean Absorbance_control wells_ – Mean Absorbance_blank wells_) × 100%.

The apoptosis detection kit was further used to evaluate the apoptosis ratio induced by the prepared nanotheranostic agent. Four groups were designed. Cells were treated with (a) PBS, (b) UCNPs@PDL, (c) UCNPs@PDL@dsDNA, and (d) UCNPs@PDL@dsDNA/DOX. After being treated with different nanoprobes for 24 h, cells was carefully collected *via* the pancreatin treatment and suspended in 1.0 mL of serum-free DMEM-containing apoptosis staining solution for 5 min. The apoptosis staining solution consisted of 195 μL binding buffer, 6.5 μL Annexin V-APC staining solution, and 6.5 μL 7-AAD staining solution. After washing with PBS several times, cells were resuspended in PBS for flow cytometry. The necessary fluorescence compensation was operated according to the manufacturer’s instructions.

### *In vivo* PDT Efficacy Assay

The animal care and handing procedures were reviewed and approved by the Animal Care and Use Committee of Linyi University. Cancer cells (100 μL, 1 × 10^6^) were injected into the right leg of balb/c nude mice (6 weeks, around 20 g) to implant the tumor. The experiment began when the tumor volume reached to 80–120 mm^3^. The tumor-bearing mice were randomly divided into four parallel groups (*n* = 5 for each group) and then received different treatments. Then, the tumor volume (V = length × width^2^/2) was recorded every 2 days over a period for 9 days and the prepared nanotheranostic agent could be injected into the tumor section on the fifth day. Finally, the mouse was sacrificed to obtain the tumor section and main organs which were sliced for hematoxylin and eosin (H&E) staining, PCNA staining, and Ki67 staining.

## Results and Discussion

### Characterization of the Prepared UCNPs@PDL Nanoprobe

**FIGURE 1:**
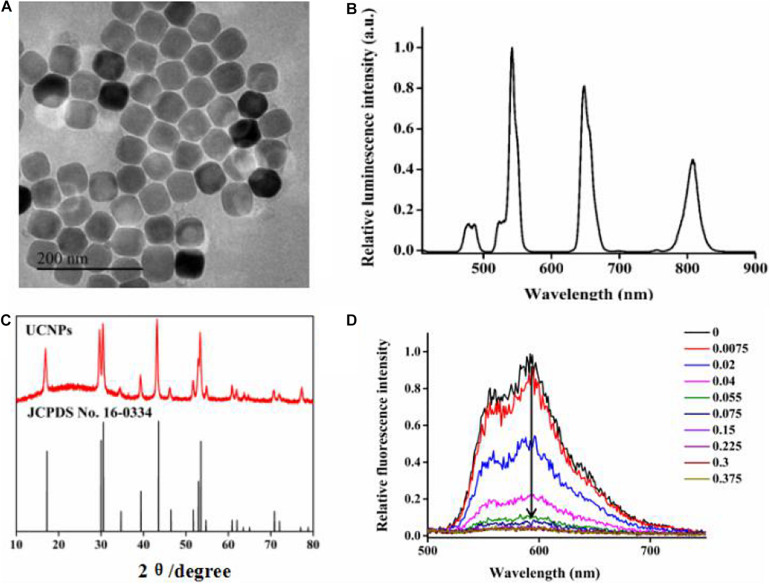
**(A)** TEM images of UCNPs, **(B)** luminescence spectra of UCNPs, **(C)** XRD images of UCNPs, and **(D)** the fluorescence spectra of 5.3 μM DOX in the HEPES buffer (20 mM, pH-7) with increasing molar ratios of the hybridized DNA duplex after incubation for 6 h.

Guided by the layer-by-layer seed-mediated shell growth strategy, the oleic acid-protected UCNPs were prepared and then treated with acidic ethanol solution to remove oleate ligands from their surface. As shown in Figure 1, the obtained UCNPs displayed homogeneous particle sizes (Figure 1A), separate luminescence peaks (Figure 1B), and a pure hexagonal phase (JCPDS No. 16-0334, Figure 1C). The cationic polymer, PDL, was uniformly coated onto the surface of bared UCNPs proved by zeta-potential analysis. As shown in Supplementary Figure 1, the modification of PDL would increase the zeta-potential of the nanoprobe from 14.1 to 40.0 mV due to the protonation effect of the surface −NH_2_ group, which was beneficial to deliver nucleic acids.

For DNA nanotrains, we designed a DNA nanotrain containing the anti-PCNA aptamer as single DNA 1 and the anti-PCNA aptamer complementary strand with AS1411 aptamer as single DNA 2 (supporting information, table S1). The formed duplex has 31 G–C base pairs suitable for DOX loading (Kotula et al., 2012; Tian et al., 2018). Furthermore, the intercalated amount of DOX into the dsDNA was evaluated by monitoring the change of DOX fluorescence intensity. When intercalated into the DNA duplex, the fluorescence intensity of DOX molecules would be sequentially decreased due to the intermolecular Förster resonance energy transfer. In addition, the decrease in degree showed a good correlation with the molar ratio of the DNA duplex. In this experiment, the molar ratio of the DNA duplex to DNA was calculated to be 0.15:1 to obtain the optimum fluorescence quenching efficacy and the best loading capacity (Figure 1D). In addition, the fluorescence intensity of the formed nanotrain did not show any obvious change when dispersed in different pH values for overnight, proving its good stability before arriving at the action site (Supplementary Figure 2).

### Characterization of the Prepared UCNPs@PDL@dsDNA/DOX Nanotheranostic Agent

In this experiment, to evaluate the therapeutic efficacy of the designed nanotrain and eliminate the interference of nanoparticles with the nucleus bioactivity, we used the UCNPs@PDL nanoprobe to physically load DNA nanotrains. Thus, the final prepared nanotheranostic agent, UCNPs@PDL@dsDNA/DOX, would be selectively endocytosed into cancer cells and then only the nanotrain further comes into the nucleus. As shown in Supplementary Figure 3A, the designed dsDNA/DOX was homogeneously coated onto the surface of the prepared UCNPs@PDL nanoprobe. The loading amount of the DNA duplex and DOX were calculated to be 2 × 10^9^ mol/mg and 1.33 × 10^–8^ mol/mg, respectively. In addition, the obtained UCNPs@PDL@dsDNA/DOX nanotheranostic agent displayed a decreased zeta potential (−20.4 mV, Supplementary Figure 3B) and an obvious characteristic Uv-Vis peak of DNA (Supplementary Figure 3C). Meanwhile, the fluorescence intensity of DOX molecules was significantly inhibited when intercalated into the DNA duplex (Supplementary Figure 3D).

### Cellular Localization and Cell Targetability of the Prepared Nanocarrier

**FIGURE 2:**
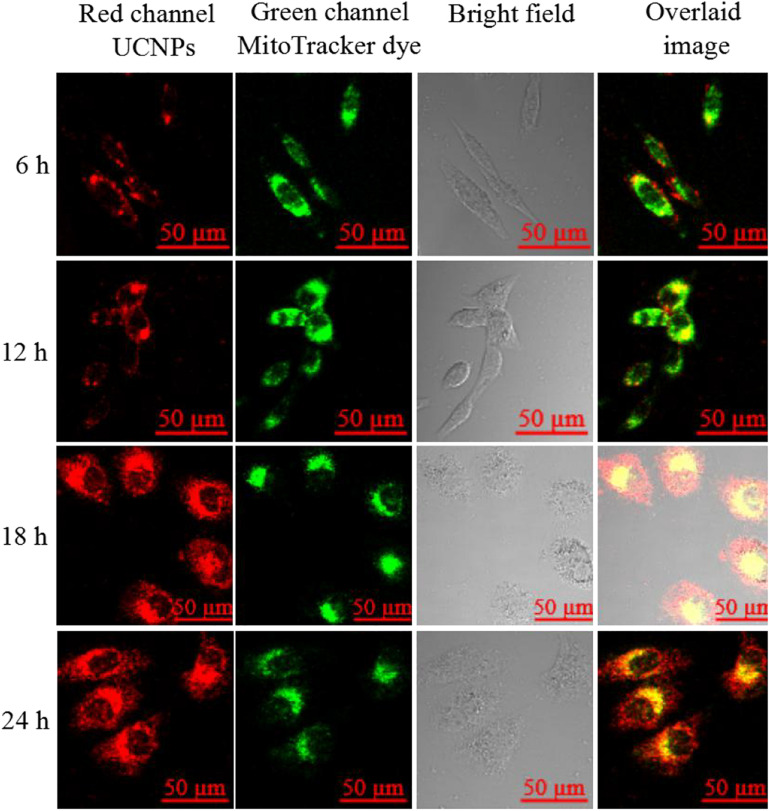
Upconversion luminescence imaging of A549 cells treated with 90 μg/mL of the prepared UCNPs@PDL@dsDNA/DOX nanoprobe. UCNP fluorescence information was collected at the red channel from 620 to 680 nm under excitation by a 980-nm laser; lysosome fluorescence information was collected at the green channel from 580 to 600 nm under the excitation at 543 nm. The overlaid image consisted of red channel and green channel.

In this experiment, to investigate the cellular localization of the finally prepared UCNPs@PDL@dsDNA/DOX nanotheranostic agent, A549 cancer cells were incubated for different times and then their upconversion luminescence information recorded with the two-photon confocal laser scanning microscope. As shown in Figure 2, a little upconversion luminescence was shown near the membrane of A549 cancer cells after being incubated for 6 h. With the incubation time prolonged to 12 h, more nanocarriers were endocytosed into the cells and then mainly localized in the lysosome which was proved by the well overlapping of the upconversion luminescence with the fluorescence of Lyso-Tracker dye. Furthermore, certain nanocarriers would escape from the lysosome and then distribute in the cytoplasm at more than 18 h of incubation time. As compared, L132 lung cells displayed negligible upconversion luminescence even incubated with the prepared nanotheranostic agent for 24 h (Supplementary Figure 4), proving that the designed aptamer-functionalized upconversion nanotheranostic agent displayed satisfactory selectivity to cancer cells and weak cytotoxicity to normal cells.

### Cellular Localization of DNA Nanotrain

**FIGURE 3:**
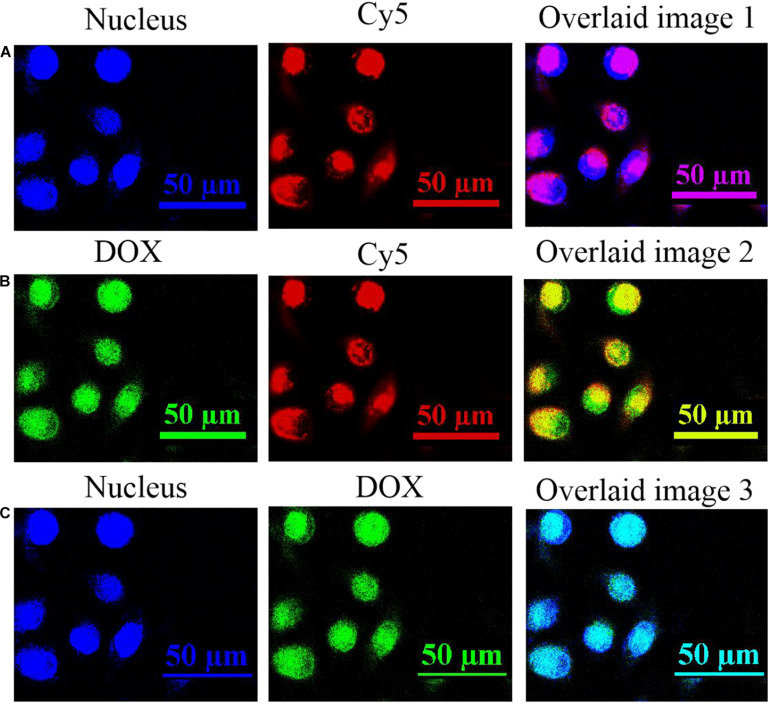
The fluorescence information in the nucleus of A549 cancer cells after being incubated with 90 μg/mL of the prepared UCNPs@PDL@dsDNA/DOX nanoprobe for 24 h. **(A)** The fluorescence images of nucleus and Cy5; **(B)** the fluorescence images of DOX and Cy5; and **(C)** the fluorescence images of nucleus and DOX. Fluorescence information of the nucleus was collected at the blue channel from 410 to 460 nm under the excitation of 405 nm; fluorescence information of DOX molecules was collected at the green channel from 525 to 620 nm under the excitation of 488 nm; fluorescence information of Cy5-labeled DNA was collected at the red channel from 640 to 720 nm under the excitation of 633 nm.

Since the DNA nanotrain was physically loaded by the UCNPs@PDL nanoprobe, its cellular localization was further studied *via* the fluorescence resonance energy transfer (FRET) strategy which used the Cy5-modified anti-PCNA aptamer and BHQ-3-modified complementary strand to establish the FRET pair. The distribution of the nucleus, anti-PCNA aptamer, and released DOX molecules in A549 cancer cells was observed with the laser confocal scanning microscope (Supplementary Figure 5). Then, separate overlaid layers were further used to clearly validate the release mechanism. As shown in Figure 3A, bright fluorescence of Cy5 was shown in the nucleus of A549 cancer cells, proving the breakup of the FRET pair due to the selective recognition and strong binding interaction of the anti-PCNA aptamer with PCNA in the nucleus. Subsequently, the separation of the DNA duplex would induce the *in situ* release of DOX molecules to the place with active PCNA, which was proved by the well-overlaid fluorescence information of DOX and Cy5 dye shown in Figure 3B. Finally, DOX molecules were distributed in the nucleus to perform their therapeutic efficacy (Figure 3C). 3D scanning further proved the release process of DOX molecules (Supplementary Figure 6). As compared, the nanotrain could not be opened in the nucleus of L132 lung normal cells due to the less-expressed nucleolin and PCNA; thus, there was no obvious release of DOX molecules in the nucleus of L132 lung normal cells (Supplementary Figure 7).

### Cytotoxicity Evaluation

**FIGURE 4:**
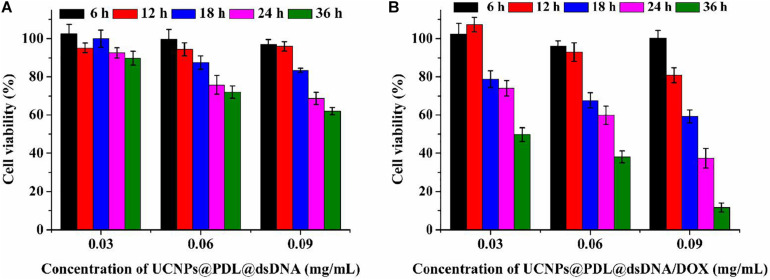
Cell viability of A549 cancer cells when treated with different concentrations of the prepared nanoprobe for different times. **(A)** UCNPs@PDL@dsDNA; **(B)** UCNPs@PDL@dsDNA/DOX.

The *in situ* release of DOX molecules into the nucleus would be proposed to perform its therapeutic efficacy well and induce significant cell apoptosis. In this experiment, the CCK-8 assay was firstly used to evaluate the cytotoxicity of the prepared nanotheranostic agent. As shown in Supplementary Figure 8, the designed UCNPs@PDL nanoprobe did not bring obvious cytotoxicity to A549 cancer cells even within 36 h, proving the good biocompatibility of UCNPs and PDL. After introducing the anti-PCNA aptamer, the prepared UCNPs@PDL@dsDNA nanoprobe could induce obvious cytotoxicity to A549 cancer cells due to the interfering effects of the anti-PCNA aptamer with DNA replication (Figure 4A). As compared, L132 normal lung cells could keep above 90% of cell viability even incubated with the UCNPs@PDL@dsDNA nanoprobe for 36 h because the specific recognition of the AS1411 aptamer and anti-PCNA aptamer would protect the surrounding healthy cells from being invaded (Supplementary Figure 9A). When DOX molecules were intercalated into the DNA duplex to prepare the final UCNPs@PDL@dsDNA/DOX nanotheranostic agent, outstanding therapeutic efficacy was displayed. The induced cytotoxicity to A549 cancer cells could reach 88.4% with 0.45 μg/mL of loaded DOX molecules, which was quite lower than the median lethal concentration (LC50) (Figure 4B). In addition, the death percentage of cancer cells could be subsequently improved with increase in the concentration and prolonged incubation time of the prepared UCNPs@PDL@dsDNA/DOX nanotheranostic agent owing to the decreased drug resistance. As compared, the cell viability of L132 normal lung cells was not obviously affected by the prepared UCNPs@PDL@dsDNA/DOX nanotheranostic agent due to the negligible release of DOX molecules (Supplementary Figure 9B). To further investigate the therapeutic efficacy, A549 cancer cells were treated with free DOX molecules with an equal amount of intercalated ones; their cell viability could keep above 72% (Supplementary Figure 10). We calculated the synergistic performance of the anti-PCNA aptamer and DOX molecules with the combination index (CI) (Mohan et al., 2001; Wu et al., 2017). In this work, around 75% of cell viability when treated for 18 h was used to calculate CI. According to Figure 4, the concentration of the designed nanotheranostic agent was 0.03 μg/mL; thus, the concentration of the loaded aptamer and DOX molecules was calculated to be 0.05 μM and 0.212 μg/mL, respectively. Meanwhile, the concentration of the prepared UCNPs@PDL@dsDNA should be around 0.12 μg/mL to reach the same cell viability which could load around 0.2 μM of aptamer. As shown in Supplementary Figure 10, 0.45 μg/mL of free DOX molecules should treat tumor cells to reach the same effect. Thus, the CI was 0.05/0.2 + 0.212/0.45 = 0.72. Thus, the anti-PCNA aptamer and DOX molecules showed synergistic effects. To be mentioned, the synergistic effects would be more obvious at the higher cell apoptosis ratio since free DOX molecules could not induce an obvious increase in the cytotoxicity with increased concentration due to the drug resistance effect (Supplementary Figure 10). Thus, the designed UCNPs@PDL@dsDNA/DOX probe was a highly selective and specific nanotheranostic agent, showing great potential in the clinical cancer treatment.

### Cell Apoptosis Evaluation

**FIGURE 5:**
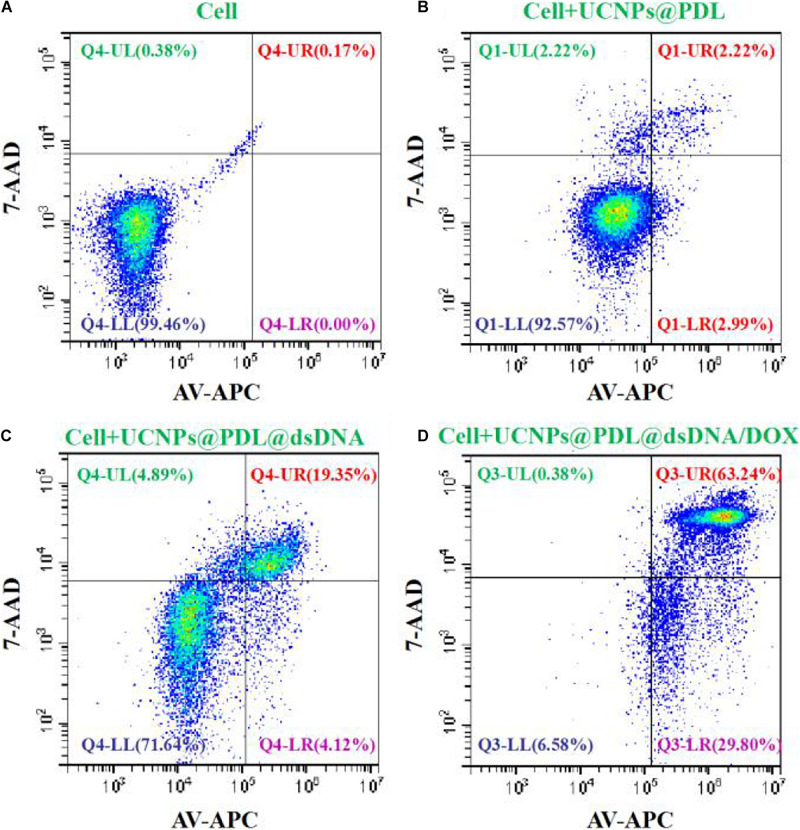
Cell viability (%) of the A549 cancer cells treated with **(A)** PBS, **(B)** UCNPs@PDL nanoprobe, **(C)** UCNPs@PDL@dsDNA nanoprobe, and **(D)** UCNPs@PDL@dsDNA/DOX nanoprobe. The fluorescence compensation was adjusted according to the manufacturers’ instructions.

The induced cell apoptosis was further evaluated by flow cytometry with the commercial Annexin V-APC/7-AAD Apoptosis Detection Kit. As shown in Figure 5, the A549 cancer cells could keep good cell viability when treated with UCNPs@PDL. After being treated with the UCNPs@PDL@dsDNA nanoprobe, around 19.35% of cancer cells were moved to the late apoptosis section. Owing to the *in situ* stimulated release of DOX molecules into the their action site, the finally prepared UCNPs@PDL@dsDNA/DOX nanotheranostic agent would induce 29.80 and 63.24% of cancer cells shifting from highly viability to early apoptosis and late apoptosis, respectively, which was higher than the ever-reported DOX involved chemotherapy (Figure 5; Li et al., 2014). L132 lung normal cells did not show obvious cell apoptosis when incubated with the prepared UCNPs@PDL@dsDNA or UCNPs@PDL@dsDNA/DOX nanotheranostic agent, proving the high selectivity of the designed nanotheranostic agent to cancer cells and its negligible side effects to normal cells (Supplementary Figure 11).

### *In vivo* Therapeutic Efficacy of the Prepared Nanotheranostic Agent

**FIGURE 6:**
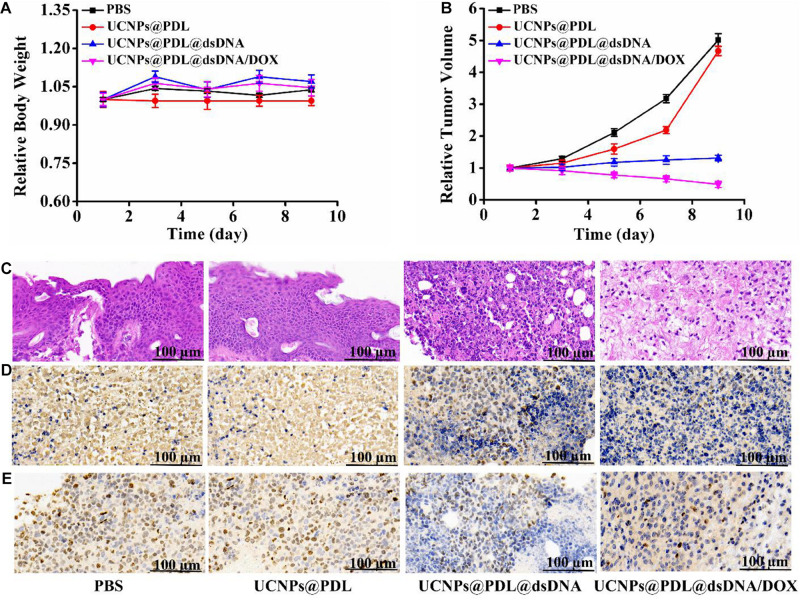
**(A)** Time-dependent mouse body weight curves of different groups of mice with various treatments. **(B)** Time-dependent tumor growth curves of different groups of mice with various treatments. **(C)** H&E staining. **(D)** PCNA staining. **(E)** Ki67 staining of the dissected tumor section on the ninth days with different treatments. The tumor-bearing mouse was treated with PBS, UCNPs@PDL nanoprobe, UCNPs@PDL@dsDNA nanoprobe, and UCNPs@PDL@dsDNA/DOX nanoprobe, respectively.

We further used the xenograft mouse model to study the *in vivo* therapeutic efficacy of the prepared nanotheranostic agent. Cancer cells (100 μL, 1 × 10^6^) were injected into the right leg of an immunodeficient mouse (balb/c nude mouse, 6 weeks, around 20 g) to culture the tumor. The experiment began when the tumor volume reached 80–120 mm^3^. The tumor-bearing mice were randomly divided into four parallel groups (*n* = 5 for each group) and then received different treatments. Group 1: injected with PBS buffer (as control group); Group 2: injected with the prepared UCNPs@PDL nanoprobe; Group 3: injected with the prepared UCNPs@PDL@dsDNA nanoprobe; and Group 4: injected with the prepared UCNPs@PDL@dsDNA/DOX nanoprobe. Then, their weight and tumor volume were measured every 2 days (Supplementary Figure 12). As demonstrated in Figure 6A, there was no significant weight change or abnormal behavior in all mouse models, proving that the experimental treatments played weak side effects. As shown in Figure 6B, the malignant tumor would remarkably grow to 4.8–5.2-fold after 9 days when treated with PBS (group 1, Figure 6B and Supplementary Figure 12). As expected, the tumor in group 2 displayed a similar increase; thus, the prepared UCNPs@PDL nanoprobe could not inhibit the proliferation of cancer cells (Figure 6B and Supplementary Figure 12). Satisfactorily, when mice were treated with the designed UCNPs@PDL@dsDNA nanoprobe (group 3), the malignant tumors just increased to 1.2–1.4-fold after treatment, proving that the anti-PCNA aptamer could effectively bind with the overexpressed PCNA in the tumor section, leading to the interfered replication of the DNA template and inhibited tumor growth (Figure 6B and Supplementary Figure 12). Furthermore, after being treated with the final obtained UCNPs@PDL@dsDNA/DOX nanotheranostic agent, mice in group 4 displayed a remarkable decrease in tumor volume which was only left to 51.3% on the ninth day (Figure 6B and Supplementary Figure 12). Therefore, the established nanotheranostic agent owned excellent therapeutic effects over conventional chemotherapy owing to the direct effects of DOX molecules on DNA replication.

### Histopathological Analysis of the Prepared Nanotheranostic Agent

After treatment, the mouse was sacrificed to obtain the tumor section and main organs for the staining analysis. Firstly, the systemic toxicity of the prepared UCNPs@PDL@dsDNA/DOX nanotheranostic agent was investigated by H&E staining. As proved in Supplementary Figure 13, the designed nanotheranostic agent did not bring any obvious tissue abnormalities or lesions to the main organs of tumor-bearing mice when treated with the prepared nanotheranostic agent, proving their negligible biotoxicity. As compared, there was an obvious nuclear shrinkage and black area in the tumor section due to the extracellular matrix of death cells which were gradually disintegrated, liquefied, and finally fused into flaky fuzzy substance without specific structures when treated with the prepared UCNPs@PDL@dsDNA and UCNPs@PDL@dsDNA/DOX nanotheranostic agent (Figure 6C; Li et al., 2014). We further used PCNA staining and Ki67 staining to investigate the effects of the established nanotheranostic agent on the PCNA activity and proliferating activity of cancer cells in the tumor section. In PCNA staining, the highly expressed PCNA in the nucleus of cancer cells could bind with the corresponding antibody and the followed DAB chromogenic agent. Thus, these nuclei would display a brown color while the PCNA-negative nucleus would display a blue color. As shown in Figure 6D, cancer cells in tumors treated with PBS or the UCNPs@PDL nanoprobe showed a high percentage of brown nuclei due to the highly expressed PCNA. As compared, when treated with the prepared UCNPs@PDL@dsDNA or UCNPs@PDL@dsDNA/DOX nanoprobe, cancer cells displayed decreased PCNA activity; thus, the stained percentage of PCNA-positive nuclei decreased and a high percentage of blue nuclei were demonstrated. Furthermore, the proliferating activities of cancer cells were evaluated with Ki67 staining. As proved in Figure 6E, a high percentage of cancer cells in the control group 1 and group 2 could reacted with the Ki67 antibody and stained with the DAB chromogenic agent to display brown nuclei, proving that the treatment of PBS or the obtained UCNPs@PDL nanoprobe would not affect the proliferating activity of cancer cells. Obviously, the amount of positive brown nuclei decreased while the negative blue nuclei increased due to the inhibited proliferating activity of cancer cells after being treated with the prepared UCNPs@PDL@dsDNA nanoprobe and UCNPs@PDL@dsDNA/DOX nanoprobe. In addition, when treated with UCNPs@PDL@dsDNA/DOX, cancer cells in the tumor section showed further decrease in proliferating activity owing to the cooperative inhibition effects of anti-PCNA aptamer and DOX molecules on the DNA replication.

## Conclusion

In summary, we have developed a novel drug delivery system, UCNPs@PDL@dsDNA/DOX, against malignancy tumors by combining UCNPs as the luminescence core, cationic polymer as the gene carrier, and aptamer as the DNA nanotrain to load DOX molecules. Under the drive of AS1411, the designed UCNPs@PDL@dsDNA/DOX nanotheranostic agent would be selectively endocytosed into nucleolin overexpressed cancer cells, then the DNA nanotrain would further come into the nucleus where the overexpressed PCNA would selectively recognize and strongly bind with the anti-PCNA aptamer to induce the *in situ* release of loaded DOX molecules. Since DOX molecules could be well distributed in the nucleus to directly interfere with DNA activity, their significant therapeutic efficacy was displayed even at the calculated concentration of 0.45 μg/mL which was quite lower than the reported LC50. As expected, the obtained nanotheranostic agent displayed a high cell apoptosis ratio (93.04%) and obvious inhibition of tumor growth (left to 51.3% after treatment). Therefore, the developed nanotheranostic agent could act as an effective drug delivery system to achieve the highly localized therapy.

## Data Availability Statement

The original contributions presented in the study are included in the article/Supplementary Material, further inquiries can be directed to the corresponding author/s.

## Ethics Statement

The animal study was reviewed and approved by the Animal Care and Use Committee of Linyi University.

## Author Contributions

XS and TY operated this work. FT performed the statistical analysis. FL, LR, and QL operated the supplementary experiments. SZ contributed the conception. All authors have read and approved this version of the article, and due care has been taken to ensure the integrity of the work.

## Conflict of Interest

The authors declare that the research was conducted in the absence of any commercial or financial relationships that could be construed as a potential conflict of interest.
