# Supplementary material for: Aptamer Functionalized Upconversion Nanotheranostic Agent With Nuclear Targeting as the Highly Localized Drug-Delivery System of Doxorubicin
Source: Front Bioeng Biotechnol. 2021 Feb 22;9:639487. doi: 10.3389/fbioe.2021.639487 (PMC7937813; doi:10.3389/fbioe.2021.639487)
Supplement: Supplementary file 1 [file Data_Sheet_1.docx]

Supplementary Material

**Aptamer Functionalized Upconversion Nanotheranostic Agent with Nuclear Targeting as the Highly Localized Drug-Delivery System of Doxorubicin**

Xinyue Song,^1,2^ Tao Yan,^1^ Feng Tian,^1^ Fengyan Li,^1^ Linlin Ren,^1^ Qiong Li,^1,2^ Shusheng Zhang^1*^

^1^Shandong Provincial Key Laboratory of Detection Technology for Tumor Markers, College of Chemistry and Chemical Engineering, Linyi University, Linyi 276005, P. R. China.

^2^Materials Science and Engineering Mobile Postdoctoral Center, Qingdao University, Shandong 266071, P. R. China.

Corresponding author email: [shushzhang@126.com](mailto:shushzhang@126.com)

**Contents**

[1. Materials 1](#_Toc44329304)

[2. Characterization Apparatus 2](#_Toc44329305)

[3. DNA Sequences 2](#_Toc44329306)

[4. Characterization Results 3](#_Toc44329307)

[4.1 Characterization results of the UCNPs@PDL nanoprobe 3](#_Toc44329308)

[4.2 Stability of the DNA nanotrain 3](#_Toc44329309)

[4.3 Characterization results of the UCNPs@PDL@dsDNA/DOX nanotheranostic agent 4](#_Toc44329310)

[5. Cell Experiment 4](#_Toc44329312)

[5.1 Cellular localization of the prepared nanocarrier to L132 normal cells 4](#_Toc44329313)

[5.2 Subcellular localization of DNA nanotrain in A549 cancer cells 5](#_Toc44329314)

[5.3 Cytotoxicity evaluation 6](#_Toc44329315)

[5.4 Cell apoptosis evaluation 8](#_Toc44329316)

[6. Animal Experiment 9](#_Toc44329318)

[6.1 Photographs of the Tumor-bearing Mice 9](#_Toc44329319)

[6.2 HE Analysis in Animal Experiment 9](#_Toc44329320)

1. **Materials**

Rare earth oxides with purities large than 99.99% were provided by Sigma-Aldrich Corp. 1-Octadecene and oleic acid were purchased from the Aladdin Reagent, Ltd. while the rest chemical reagents were obtained from Sinopharm Chemical Reagent Co., Ltd. (Shanghai, China). All sample solution was prepared with ultrapure water provided by an OKP purification system. The animal care and use committee of Linyi university reviewed and supervised the animal care and handing procedures. The commercial fluorescent dye was purchased from the Beyotime Biotechnology Co., Ltd. (Shanghai, China). The DNA strands were synthesized and provided by the Sangon Biotech Co., Ltd. (Shanghai, China).

1. **Characterization Apparatus**

The prepared nanoparticles was analyzed with the transmission electron microscope (TEM, model: JEM-2010, JEOL) and X-ray powder diffractometer (XRD, model: D8 ADVANCE) to characterize the size, morphology and crystalline forms. The luminescence spectra of the prepared UCNPs was excited by an external 980 continuous-wave laser and then recorded by a fluorescence spectrophotometer (mode: F-4600, Hitachi). The UV-Vis spectrophotometer (model: Cary 60, Agilent) was used to record the UV-Vis absorption spectra and the FT-IR spectrophotometer was used to obtain the FT-IR spectra. The ζ potential of the nanoprobes were analyzed by the Zeta-size nano instrument (Zen 3600, Malvern Instruments Ltd.). The confocal laser scanning microscope (Nikon, A1+) and two-photon confocal laser scanning microscope (Leica TCS SP5) were used to record the fluorescent images. The microplate reader (Thermo Scientific Multi-skan Mk3) was used to analyze the CCK-8 cytotoxicity results and the flow cytometry (Beckman Coulter, Inc.) was used to collect the cellular average fluorescence information.

1. **DNA Sequences**

Table 1: DNA sequences of AS1411 aptamer, DNA-1, DNA-2, fluorescence labeled DNA-1 and quencher labeled DNA-2.

| DNA strands | 5' to 3' | modifier |
| --- | --- | --- |
| AS1411 aptamer | GGT GGT GGT GGT TGT GGT GGT GGT GG |  |
| DNA-1  (Anti-PCNA aptamer) | CAT GCT TCC CCA GGG AGA TGC CTA TGG TCC CCG CGT AGG TGG CAG CTC A | No |
| DNA-2 | TGA GCT GCC ACC TAC GCG GGG ACC ATA GGC ATC TCC CTG GGG AAG CAT GGG TGG TGG TGG TTG TGG TGG TGG TGG | No |
| Cy5 modified DNA-1 | CAT GCT TCC CCA GGG AGA TGC CTA TGG TCC CCG CGT AGG TGG CAG CTC A | 5’ modified with Cy5 |
| Quencher modified DNA-2 | GGT GGT GGT GGT TGT GGT GGT GGT GGT GAG CTG CCA CCT ACG CGG GGA CCA TAG GCA TCT CCC TGG GGA AGC ATG | 3’ modified with BHQ-3 |

1. **Characterization Results**

**4.1 Characterization results of the UCNPs@PDL nanoprobe**


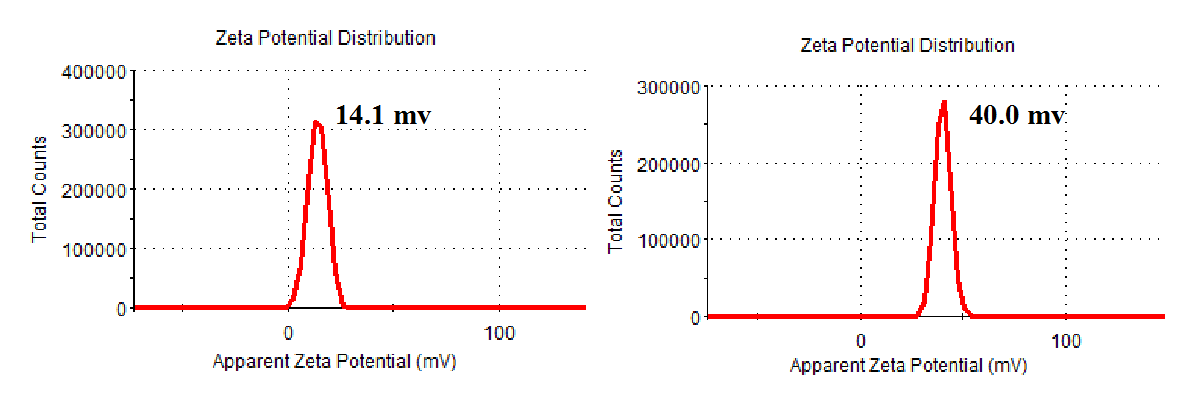
The modification of PDL onto the surface of bared UCNPs was analyzed by the Zeta-potential analysis. As shown in Figure S1, the Zeta-potential of the nanoprobe increased from 14.1 mV to 40.0 mV due to the protonation effect of the surface -NH_2_ group.

Figure S1: Zeta-potential of (a) bared UCNPs and (b) UCNPs@PDL.

**4.2 Stability of the DNA nanotrain**


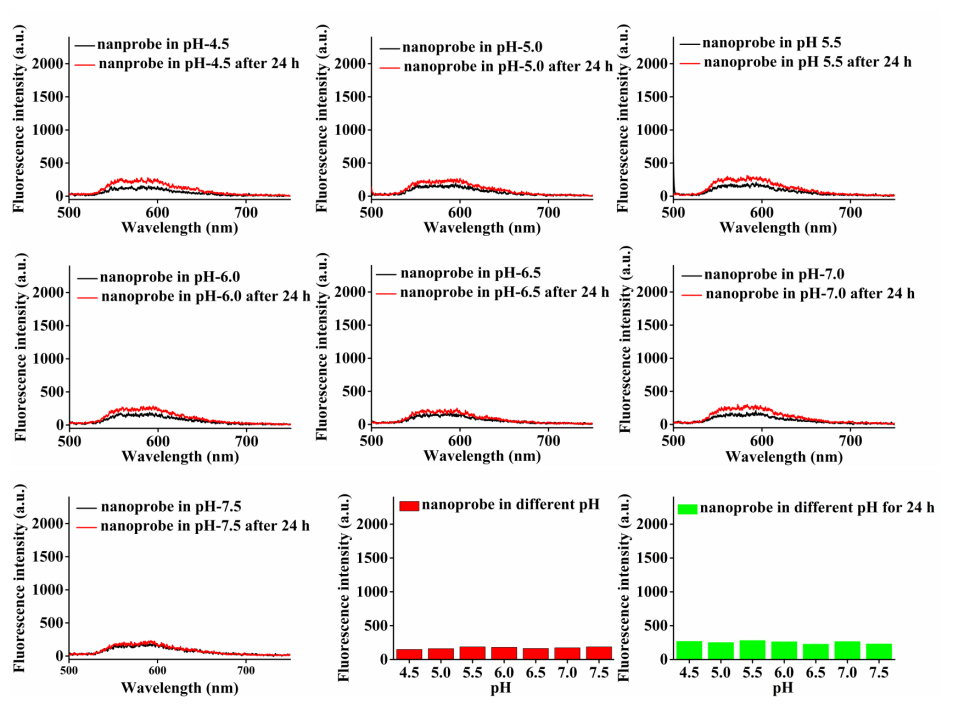


Figure S2: Fluorescence spectra of dsDNA/DOX (1.0 µM) dispersed in different pH value for overnight.

**4.3 Characterization results of the UCNPs@PDL@dsDNA/DOX nanotheranostic agent**

As shown in Figure S3a, the designed dsDNA/DOX was homogeneously coated onto the surface of the prepared UCNPs@PDL nanoprobe. In addition, the obtained UCNPs@PDL@dsDNA/DOX nanotheranostic agent displayed decreased zeta-potential (-20.4 mV, Figure S3b) and obvious characteristic Uv-Vis peak of DNA (Figure S3c). Meanwhile, the fluorescence intensity of DOX molecules was significantly inhibited when intercalated into the DNA duplex (Figure S3d).

**
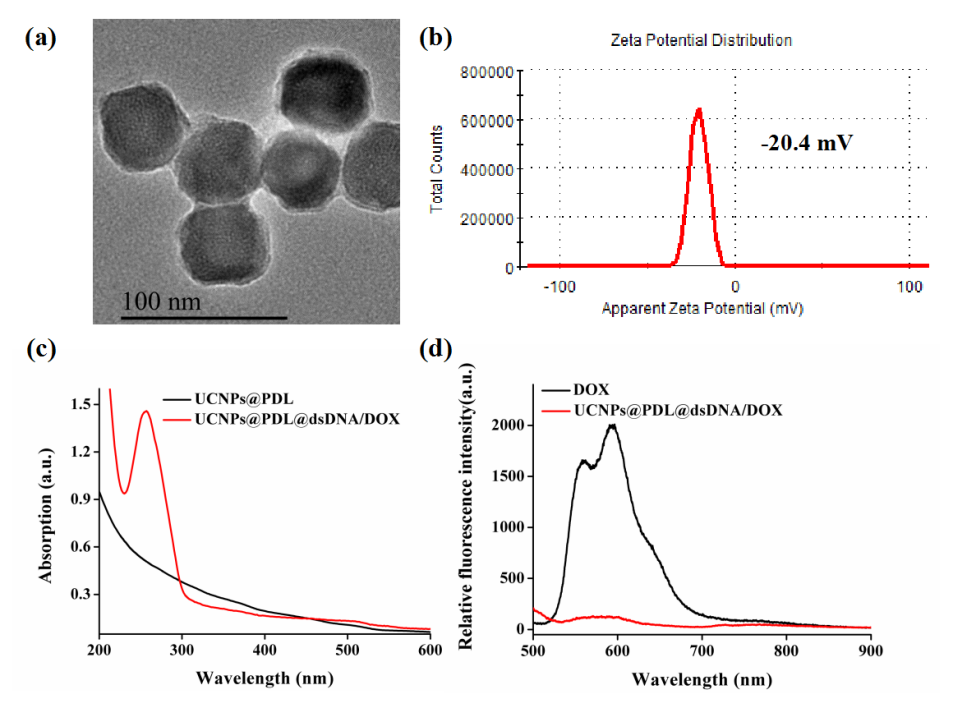
**

Figure S3: (a) TEM of the prepared UCNPs@PDL@dsDNA/DOX nanoprobe; (b) zeta-potential of the prepared UCNPs@PDL@dsDNA/DOX nanoprobe; (c) Uv-Vis spectra of the prepared UCNPs@PDL and UCNPs@PDL@dsDNA/DOX nanoprobe; (d) fluorescence spectra of free DOX and the prepared UCNPs@PDL@dsDNA/DOX nanoprobe.

1. **Cell Experiment**
   1. **Cellular localization of the prepared nanocarrier to L132 normal cells**

The prepared nanotheranostic agent was used to incubate L132 normal lung cells for different times. As shown in Figure S4, the L132 lung cells displayed negligible upconversion luminescence even incubated for 24 h. Thus, the prepared nanocarrier displayed weak cytotoxicity to normal cells.


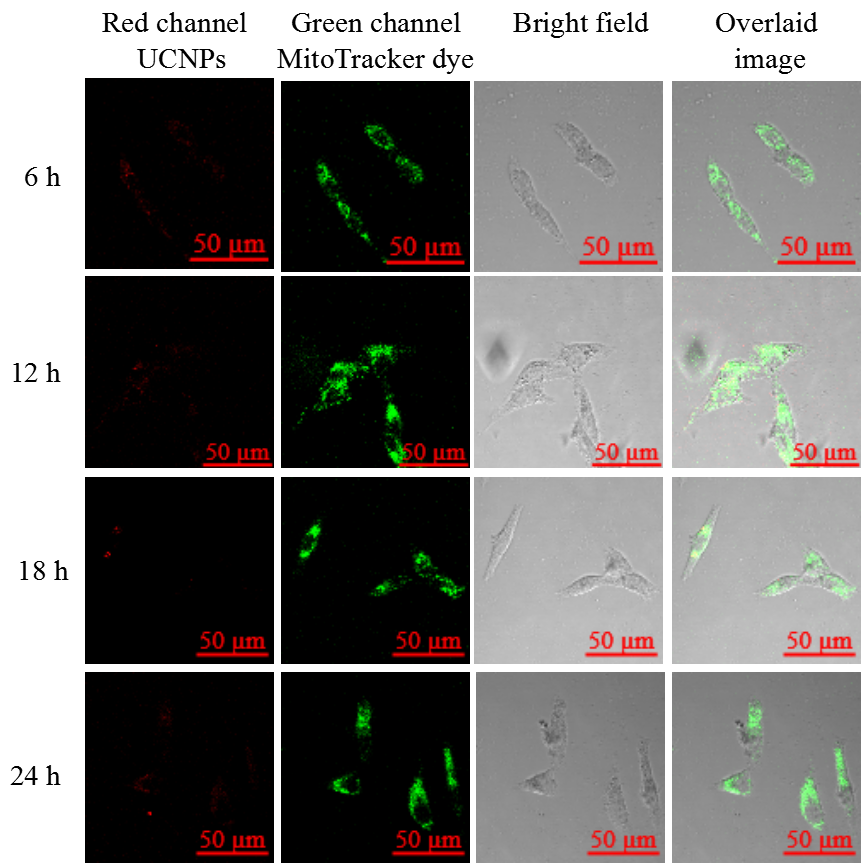


Figure S4: Upconversion luminescence imaging of L132 normal lung cells treated with 90 µg/mL of the prepared UCNPs@PDL@dsDNA/DOX nanoprobe. The UCNPs fluorescence information was collected at the red channel from 620 nm to 680 nm under the excitation of NIR laser; the lysosome fluorescence information was collected at green channel from 580 nm to 600 nm under the excitation of 543 nm. Overlaid image consisted of green channel and bright field.

**5.2 Subcellular localization of DNA nanotrain in A549 cancer cells**

As shown in Figure S5 and Figure S6, over-expressed PCNA protein of cancer cells would selectively attach to anti-PCNA aptamer, leading to the *in-situ* release of DOX molecules into nucleus.


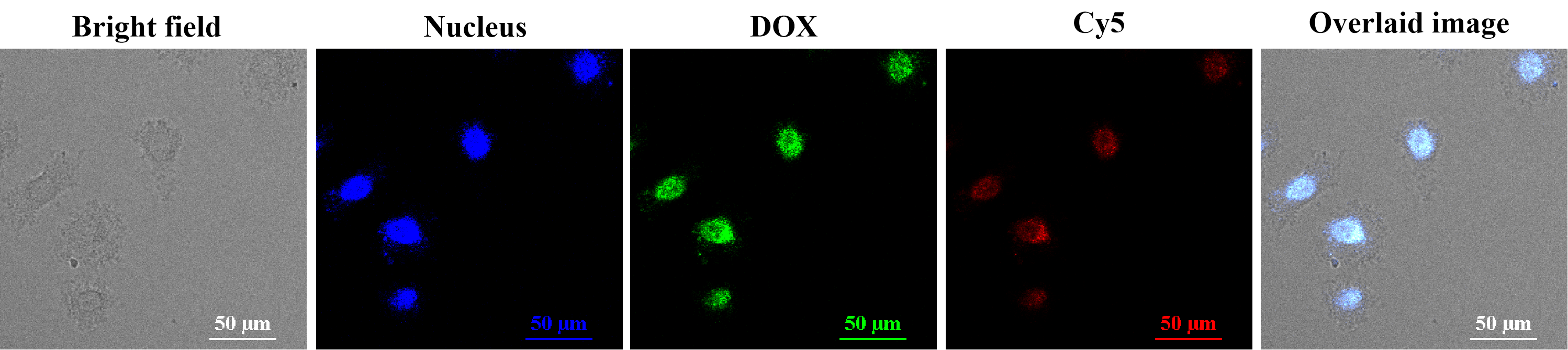
Figure S5: The fluorescence information in nucleus of A549 cancer cells after incubated with 90 µg/mL of the prepared UCNPs@PDL@dsDNA/DOX nanoprobe for 24 h. The fluorescence information of nucleus was collected at the blue channel from 410 nm to 460 nm under the excitation of 405 nm; the fluorescence information of DOX molecules was collected at the green channel from 525 nm to 620 nm under the excitation of 488 nm; the fluorescence information of Cy5 labeled DNA was collected at the red channel from 640 nm to 720 nm under the excitation of 633 nm.


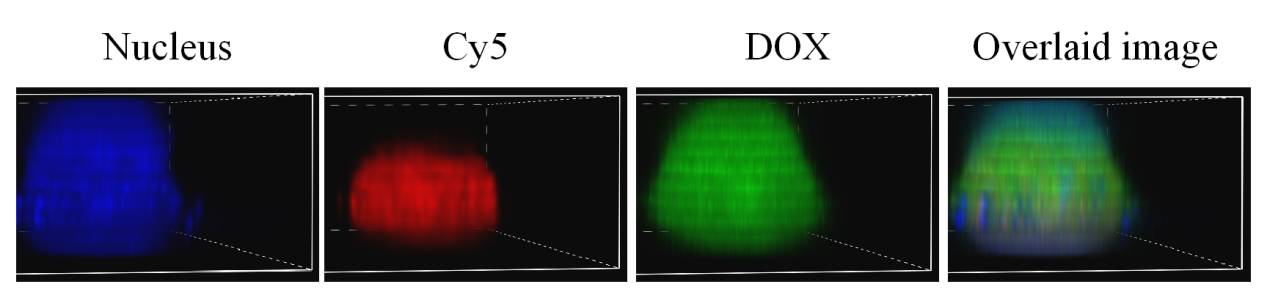


Figure S6: 3D scanning of single cancer cells. The nucleus fluorescence information was collected at the blue channel from 410 nm to 460 nm under the excitation of 405 nm; the DOX fluorescence information was collected at the blue channel from 525 nm to 620 nm under the excitation of 488 nm. The fluorescence information of Cy5 labeled DNA was collected at the red channel from 640 nm to 720 nm under the excitation of 633 nm. Overlaid image consisted of nucleus, DOX and Cy5.

As shown in Figure S7, the nanotrain could not be opened in the nucleus of L132 lung normal cells due to the less-expressed nucleolin and PCNA. Therefore, there was not obvious release of DOX molecules.


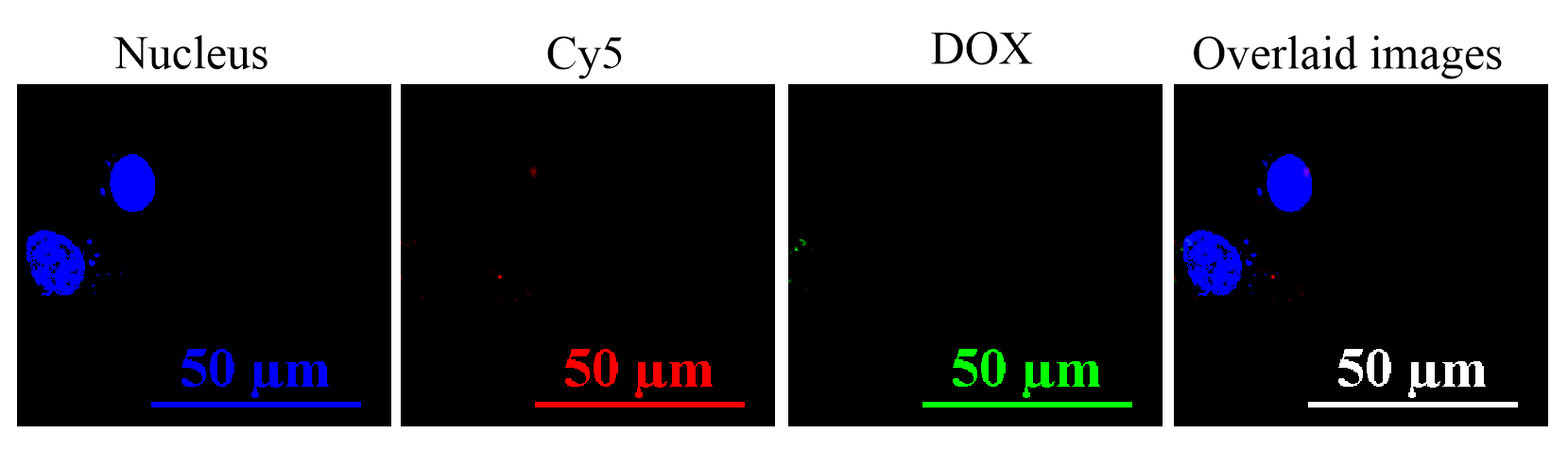


Figure S7: Fluorescence information in the nucleus of L132 lung normal cells after incubated with the prepared UCNPs@PDL@dsDNA/DOX nanoprobe for 24 h. Fluorescence images of nucleus, Cy5, DOX and overlaid image.

**5.3 Cytotoxicity evaluation**

As demonstrated in Figure S8, different concentrations of the prepared UCNPs@PDL nanoprobe (0-90 µg/mL) did not induce obvious cytotoxicity of the A549 cancer cells.


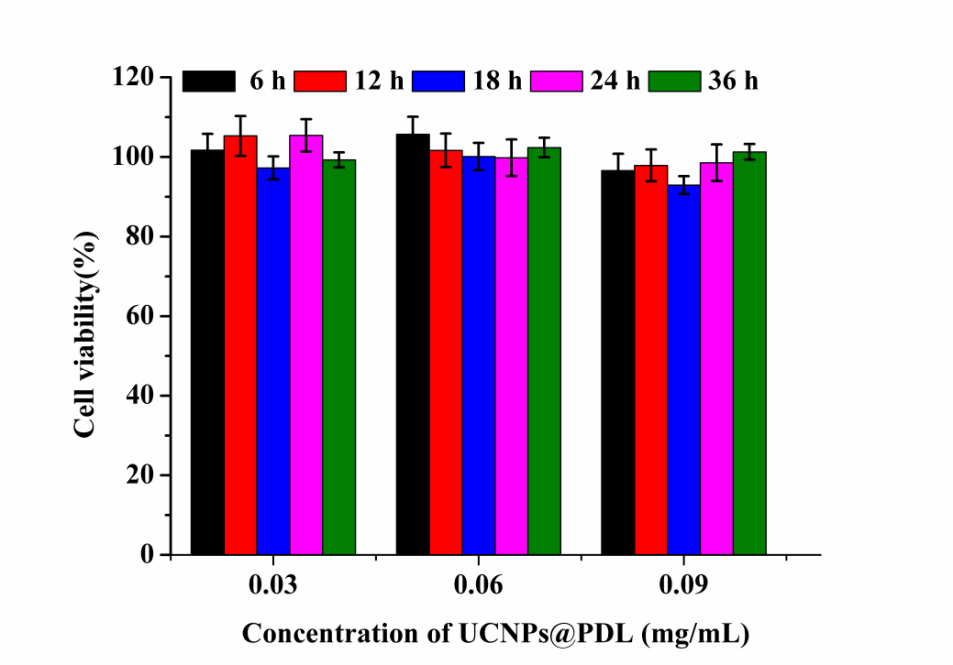


Figure S8: Cell viability (%) under different concentrations of the UCNPs@PDL nanoprobe. Each concentration was operated five times and error bars represent standard deviation (n=5).

To evaluate its side effects, the cytotoxicity of the prepared nanoprobe to L132 normal lung cells was further tested with CCK-8 assay. As demonstrated in Figure S9, the L132 normal lung cells could keep above 90% of cell viability when incubated with the prepared UCNPs@PDL@dsDNA or UCNPs@PDL@dsDNA/DOX nanoprobe even for 36 h. Thus, the designed nanotheranostic agent owned high selectivity and specificity, showing great potentials in clinical cancer treatment.


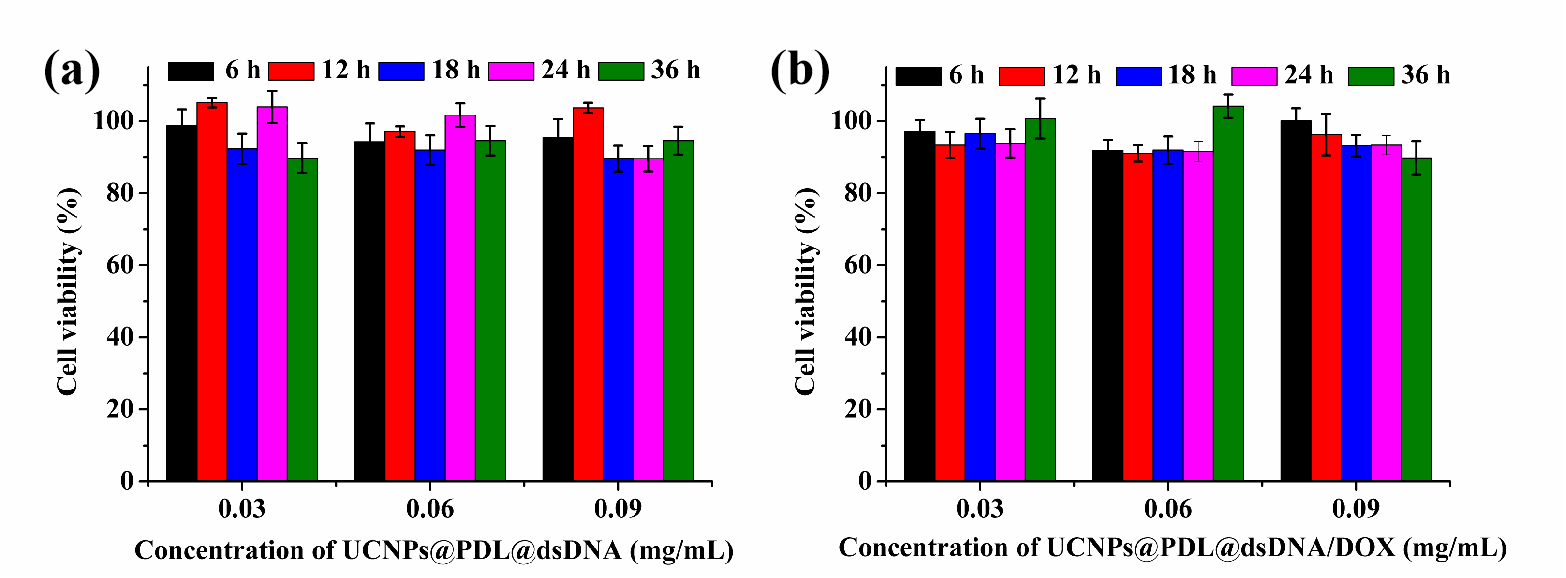
Figure S9: The cell viability (%) of L132 lung normal cells when incubated with different concentrations of the prepared nanoprobe for different times. (a) UCNPs@PDL@dsDNA; (b) UCNPs@PDL@dsDNA/DOX. Each concentration was operated five times and error bars represented standard deviation (n=5).

When treated with 0.15-0.45 µg/mL of DOX molecules for different times, their cell viability could keep above 72% (Figure S10).


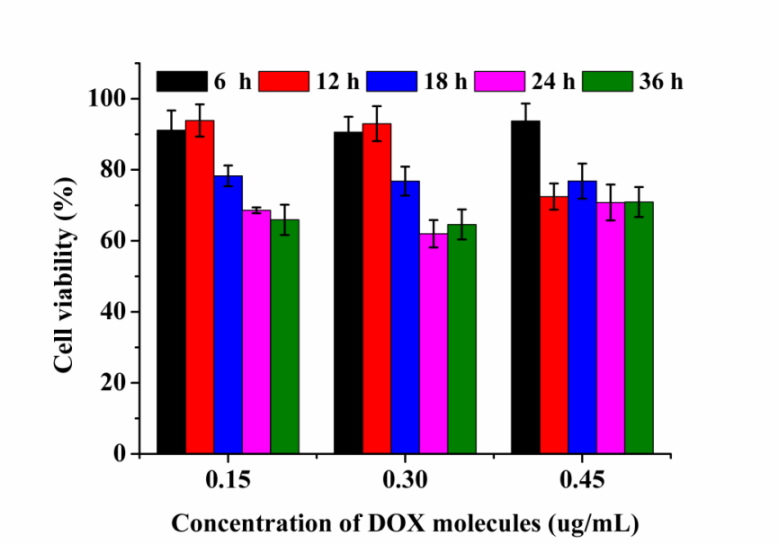


Figure S10: The cell viability (%) of A549 cancer cells when incubated with different concentrations of DOX molecules for different times. Each concentration was operated five times and error bars represented standard deviation (n=5).

**5.4 Cell apoptosis evaluation**

As shown in Figure S11, the prepared UCNPs@PDL@dsDNA or UCNPs@PDL@dsDNA/DOX nanotheranostic agent did not induce obvious cell apoptosis to L132 lung normal cells.


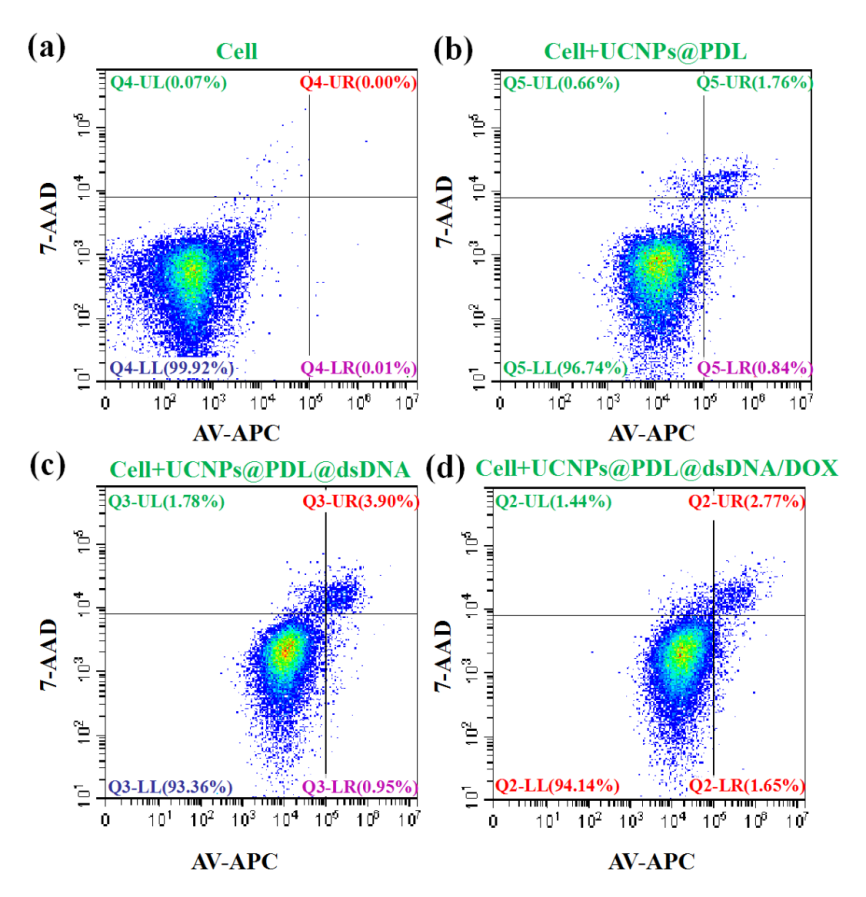


Figure S11: Cell viability (%) of the L132 lung normal cells treated with (a) PBS, (b) UCNPs@PDL nanoprobe, (c) UCNPs@PDL@dsDNA nanoprobe, (d) UCNPs@PDL@dsDNA/DOX nanoprobe. The fluorescence compensation was adjusted according to the instrument.

1. **Animal Experiment**
   1. **Photographs of the Tumor-bearing Mice**

**
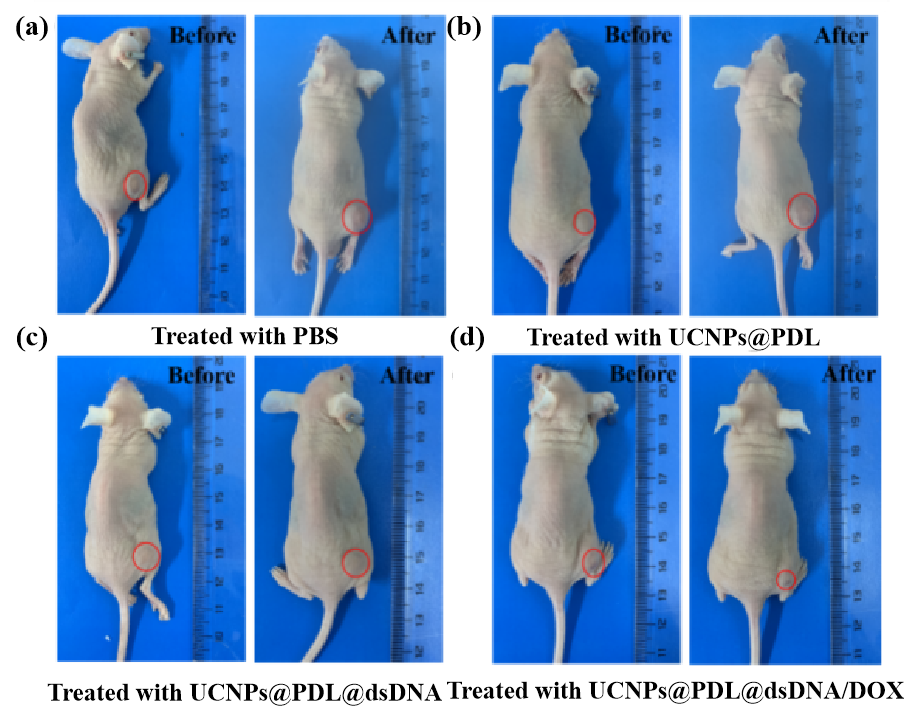
**As shown in Figure S12, the malignant tumor remarkably grow to 4.8-5.2 fold after 9 days after treated with PBS. As expected, the tumor displayed the similar increase trendency after treated with the UCNPs@PDL nanoprobe. Satisfactorily, when mice were treated with the designed UCNPs@PDL nanoprobe. Satisfactorily, when mice were treated with the designed UCNPs@PDL@dsDNA nanoprobe, their malignant tumors just increased to 1.2-1.4 fold. Furthermore, the tumor volume displayed remarkably decrease which was only left to 51.3% on the ninth day after treated with the final obtained UCNPs@PDL@dsDNA/DOX nanotheranostic agent.

Figure S12: Photographs of the tumor-bearing mice on the first day and the ninth day after being received with different treatments.

- 1. **HE Analysis in Animal Experiment**

**
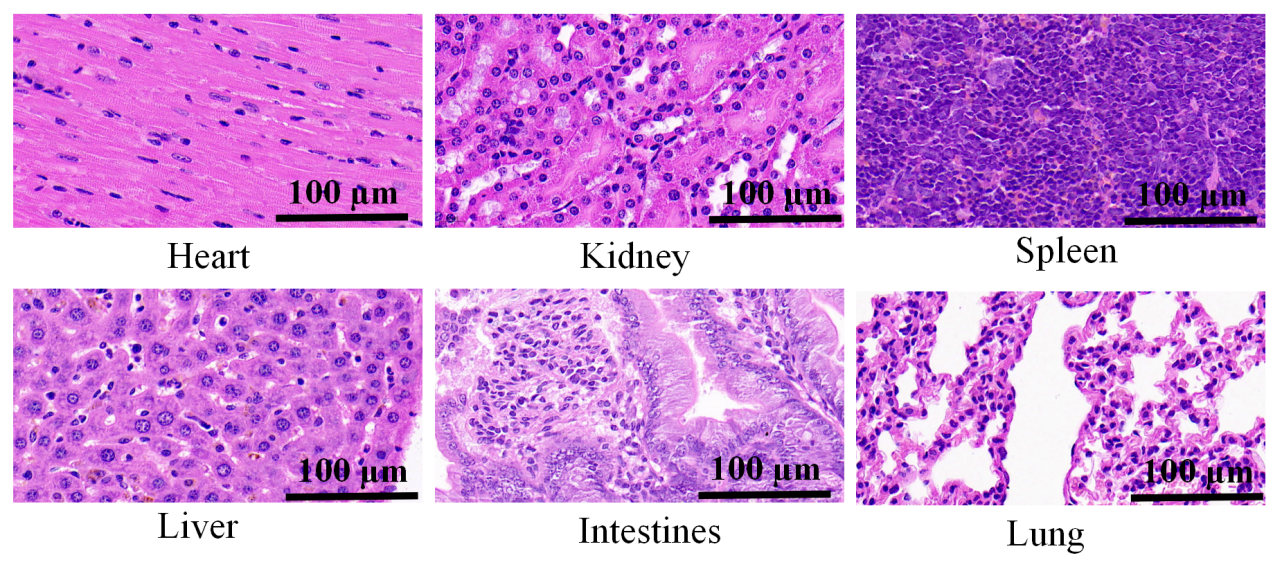
**As shown in Figure S13, the designed nanotheranostic agent did not bring any obvious tissue abnormalities or lesions to the main organs of the tumor-bearing mouse when treated with the prepared nanotheranostic agent, proving their negligible biotoxicity.

Figure S13: Bio-toxicity investigation of the prepared UCNPs@PDL@dsDNA/DOX nanotheranostic agent to the main organs.
